# Advances in the Treatment of Relapsed and Refractory Multiple Myeloma in Patients with Renal Insufficiency: Novel Agents, Immunotherapies and Beyond

**DOI:** 10.3390/cancers13205036

**Published:** 2021-10-09

**Authors:** Boris Bozic, Jens Rutner, Chang Zheng, Reinhard Ruckser, Flonza Selimi, Krysztina Racz, Martin Köcher, Georg Tatzreiter, Christian Sebesta

**Affiliations:** 1 2nd Medical Department, Hematology and Oncology, Klinik Donaustadt, 1220 Vienna, Austria; jens.rutner@gesundheitsverbund.at (J.R.); chang.zheng@gesundheitsverbund.at (C.Z.); reinhard.ruckser@gesundheitsverbund.at (R.R.); flonza.selimi@gesundheitsverbund.at (F.S.); krysztina.racz@gesundheitsverbund.at (K.R.); martin.koecher@gesundheitsverbund.at (M.K.); georg.tatzreiter@gesundheitsverbund.at (G.T.); christian.sebesta@gesundheitsverbund.at (C.S.); 2 Donau-Science, 1220 Vienna, Austria

**Keywords:** relapsed and refractory multiple myeloma, renal insufficiency, novel agents

## Abstract

**Simple Summary:**

The treatment of patients with relapsed/refractory multiple myeloma has advanced considerably in recent years, irrespective of whether they are younger or older patients. Treating patients with relapsed/refractory multiple myeloma and renal impairment is far more complicated than treating patients with normal renal function due to the frequent dose adjustments that are necessary and the resulting loss in effectiveness.

**Abstract:**

Background: Renal insufficiency is one of the most frequent complications in multiple myeloma. The incidence of renal insufficiency in patients with multiple myeloma ranges from 20% to 50%. Renal impairment in patients with multiple myeloma results primarily from the toxic effects of monoclonal light chains on the kidneys. Dehydration, hypercalcemia, hyperuricemia, the application of nephrotoxic NSARs, antibiotics, contrast agents, etc., all play a major role in the deterioration of renal function in patients with multiple myeloma. The diagnosis and treatment of these patients use an interdisciplinary approach in consultation with hematologist–oncologists, radiologists, nephrologists and intensive care specialists. Using new drugs in the treatment of patients with refractory/relapsed multiple myeloma and renal insufficiency markedly improves progression-free survival and overall survival in these patients. Conclusions: New drugs have helped to widen the treatment options available for patients with renal impairment and refractory/relapsed multiple myeloma, since dose adjustments are unnecessary with carfilzomib as well as with panobinostat, elotuzumab, pomalidomide or daratumumab in patients with renal impairment. Several new substances for the treatment of refractory/relapsed multiple myeloma have been approved in the meantime, including belantamab mafodotin, selinexor, melflufen, venetoclax, CAR T-cell therapy and checkpoint inhibitors. Ongoing studies are investigating their administration in patients with renal impairment.

## 1. Introduction

Renal impairment is defined as serum creatinine (sCr) above the upper normal limit of 2 mg/dL or as an estimated glomerular filtration rate (eGFR) of 60 mL/min/1.73 m^2^ [1]. Patients with r/r MM (relapsed and refractory multiple myeloma) present three different clinical pictures: (1) relapsed but not refractory, (2) relapsed and refractory and (3) primary refractory r/r MM [2].

Renal impairment may develop over time. It is estimated that 25 to 50% of multiple myeloma patients are affected in the course of their disease [3].

Relapsed multiple myeloma is defined as a progressive disease that occurs when a patient no longer responds to a previous therapy or requires a salvage therapy but does not yet meet the criteria ’primary refractory’ or ’relapsed and refractory’ based on the following laboratory and radiological findings: 25% increase on the lowest confirmed response in light of one or several of the following criteria: serum M protein (absolute increase must be 0.5 g/dL); serum M protein increases by 1 g/dL, when the lowest M component amounted to 5 g/dL; urine M protein (absolute increase must amount to 200 mg/24h). In patients without measurable serum and urine m protein levels, the difference between involved and uninvolved FLC levels (absolute increase must be >10 mg/dL). Occurrence of a new lesion (en), 50% increase from nadir in SPD of >1 lesions or 50% increase in the longest diameter of a previous lesion >1 cm in the short axis; 50% increase in circulating plasma cells (at least 200 cells per litre) if this is the only measure for the disease. To qualify as a clinical relapse, one or several of the following criteria must be met:

− A marked increase in the size of existing plasmacytomas or bone lesions. A definitive increase is defined as a 50% increase (and 1 cm) serially measured by the SPD of the measurable lesion;
− Hypercalcemia (>11 mg/dL);
− A decrease of 2 g/dL in haemoglobin, with no link between the decrease and the therapy or any other states not induced by the myeloma;
− Increase in serum creatinine by 2 mg/dL or more at the start of the therapy and attributable to the myeloma;
− Hyperviscosity in connection with the serum paraprotein;
− Development of new soft tissue plasmacytomas or bone lesions (osteoporotic fractures do not present a progression) [3];

Primary refractory multiple myeloma:

Patients are considered primary refractory when they have attained stable disease (SD) or progressive disease (PD) in first-line therapy [4].

## 2. The Pathophysiology of Renal Insufficiency in Multiple Myeloma

Renal failure in patients with MM results from the toxic effects of monoclonal light chains on renal structures, primarily the renal tubules. Light chain cast nephropathy occurs most often in MM patients with renal impairment. Therefore, light chain cast nephropathy is the most common diagnosis in patients with MM and significant renal insufficiency. A nephrotic syndrome occurs much more frequently in patients with amyloidosis or MIDD (monoclonal immunoglobulin deposition disease, ‘Randall disease’) (e.g., severe albuminuria, hypoalbuminemia in the serum and edemas) [5].

Hypercalcemia, dehydration, nephrotoxic drugs (aminoglycoside antibiotics and/or NSAR) and contrast agents contribute to the development or progression of preexisting RI by amplifying the toxic effect of light chains [6]. Cast nephropathy occurs when light chain production overcomes the capacity of tubular cells to endocytose and catabolize the filtered, free light chains. As a result, excess light chains form aggregates and casts with uromodulin in the distal nephron, leading to tubular obstruction and concomitant inflammation [7,8].

## 3. Novel Agents in the Therapy of Relapsed/Refractory Multiple Myeloma

The therapy options available for the treatment of multiple myeloma have improved considerably in the past 15 years once IMiDs and proteasome inhibitors had been approved. Despite the many different therapy combinations, most patients experience a relapse. In recent years, different active agents with varying mechanisms of action and distinct objectives have been studied, including cellular therapies, monoclonal antibodies and small molecules, in order to significantly prolong survival [9]. In recent years, many new drugs have been approved: pomalidomide, iberdomide, carfilzomib, ixazomib, marizomib, oprozomib, panobinostat and the three monoclonal antibodies daratumumab, isatuximab (which targets CD38) and elotuzumab. In addition, further substances have been developed which can be administered against lenalidomide-refractory as well as relapsed and refractory disease after two or more previous lines of therapy, such as monoclonal antibodies targeting BCMA, monoclonal antibodies targeting APRIL, monoclonal antibodies targeting immune checkpoints, DNA damaging agents (melflufen), inhibitors of BCL2 family proteins (venetoclax), MCL1 inhibitors, epigenetic inhibitors (histone deacetylase HDAC panobinostat) and inhibitors of nuclear cytoplasmic transport receptors, such as XPO1 inhibitor (selinexor). See Table 1.

### 3.1. IMiDs

#### 3.1.1. Thalidomide

Renal function has been shown not to influence the pharmacokinetics of thalidomide [10]. Thalidomide therapy in patients with renal failure or renal impairment does not increase the occurrence of adverse events. Therefore, the thalidomide dose does not need to be reduced for patients with renal impairment. No dose adjustment is needed, neither for renal nor for hepatic insufficiency. The dose does not need to be changed during haemodialysis. As thalidomide is known to have thrombogenic properties, the administration of prophylactic anticoagulation should be considered for patients with a dialysis shunt in order to prevent shunt thombrosis [11]. However, thalidomide is poorly tolerated in elderly patients.

#### 3.1.2. Lenalidomide

Lenalidomide is a second-generation immunomodulatory drug (IMiD) used to treat patients with relapsed or refractory myeloma. Despite primary excretion by the kidney, a dose-adjusted treatment using lenalidomide is an effective therapy option for patients with MM and renal impairment [12]. PrE1003, a PrECOG study, has also demonstrated the potential to continuously dose patients suffering from severe renal insufficiency (CrCl < 30 but not on dialysis—group B) with at least 15 mg and possibly 25 mg of lenalidomide, although the number of patients treated was too small to deliver conclusive results. Overall, this simplifies the dosing to daily dosing in all patients irrespective of renal function, with the caveat that for patients with severe renal insufficiency the dose may have to be reduced to 15 mg [13]. Oehrlin collected the data of 26 patients undergoing therapy at four different German centres and, in a substantial proportion of the cases, showed improved renal function in patients with relapsed and/or refractory MM and renal impairment after treatment using lenalidomide/dexamethasone-based therapy schemata [14].

#### 3.1.3. Pomalidomide

Pomalidomide is an IMiD developed after thalidomide and lenalidomide. Before being excreted, pomalidomide is largely metabolised by CYP450 in the liver, and unlike lenalidomide only 2% of the pomalidomide that has not been metabolised is excreted in urine [15]. The results of the MM-013 study show that patients with RRMM and moderate or severe RI, including those requiring haemodialysis, benefit from a therapy with pomalidomide in addition to low-dose dexamethasone, with ORRs of 39.4%. A daily pomalidomide dose of 4 mg plus low-dose dexamethasone is effective in patients with RRMM and moderate or severe RI, including patients at an advanced stage of the disease that require haemodialysis. The safety profile between the three groups was acceptable, and no new safety signals were observed [16]. As a third-line therapy, pomalidomide plus low-dose dexamethasone is safe and effective in patients with RRMM in whom lenalidomide-based therapy has failed; this is a clinically relevant patient population that is poorly represented in clinical studies [17].

#### 3.1.4. Iberdomide

Iberdomide is a novel, orally administered and highly effective cereblon modulator [18]. The combination of IberDd (iberdomide plus daratumumab and dexamethasone) and IberVd (iberdomide plus bortezomib and dexamethasone) exhibits a favourable tolerability profile in patients with heavily pretreated RRMM with promising clinical activity; the same applies to patients who were refractory to their previous regimen and have previously been exposed to IMiD agents, proteasome inhibitors and CD38 antibodies. Immune profiling data confirm that iberdomide and dexamethasone were pharmacodynamically active in a triplet combination and not augmented by the addition of daratumumab or bortezomib. This study is being continued for both cohorts with continued enrolment at a dose level of 1.6 mg. Updated findings, including for MTD/RP2D, will be presented. These results support further development of iberdomid-based therapies in patients with MM; phase three studies are planned to evaluate these combinations [19]. These studies evaluate the safety and tolerance of iberdomide in patients with renal impairment versus patients with normal renal function (study NCT04933747).

### 3.2. Proteasome Kinases Inhibitors

#### 3.2.1. Bortezomib

Bortezomib is active and well-tolerated in patients with renal impairment, including those who require dialysis [20]. The pharmacokinetics of bortezomib are not affected by the degree of renal impairment as the primary metabolic pathway of bortezomib is oxidative deboronation by hepatic cytochrome P450 enzymes [21]. Bortezomib should be given after dialysis in the patients who are dialysed. Ludwig et al. reported the reversal of light-chain- induced acute renal failure with bortezomib-based therapy in five out of eight patients with MM. These patients received chemotherapy with a bortezomib-doxorubicin-dexamethasone regimen. Furthermore, other bortezomib-based regimens, such as bortezomib-cyclophosphamide-dexamethasone, can also be used [21]. In the HOVON65/GMMG-HD4 trial, bortezomib resulted in a superior outcome in patients with increased serum creatinine. In these patients, both median PFS (13 vs. 30 months; hazard ratio HR, 0.45; 95% CI, 0.26–0.78; and *p* = 0.004) and OS (21 vs. 54 months; HR, 0.33; 95% CI, 0.16–0.65; and *p* = 0.001) dramatically improved with bortezomib compared to thalidomide. Bortezomib maintenance after HDT and ASCT significantly improved the nCR plus CR rate from 31 to 49% [22]. Bortezomib at the standard dose of 1.3 mg m^2^ should be considered an appropriate treatment option for the sizeable proportion of patients with relapsed MM who have any degree of renal impairment [23].

#### 3.2.2. Carfilzomib

Carfilzomib is a tetrapeptide epoxyketone PI that irreversibly binds to the β5-proteasome subunit and the LMP7 (iβ5) subunit of the immunoproteasome with greater affinity than bortezomib [24]. A phase two study comprising patients with MM and varying degrees of renal impairment showed no difference in carfilzomib clearance or exposure (15 or 20 mg/m^2^) between patients with normal renal function and patients with varying degrees of renal impairment, including patients with terminal renal insufficiency (ESRD) [25]. In the ENDEAVOUR study, carfilzomib showed improved PFS and OS compared to bortezomib in patients with varying CrCL values, including in patients with severe renal impairment (CrCl < 15 to 50 mL/min). In all treatment groups, patients who reached complete renal remission exhibited better PFS and OS outcomes than non-responders. These results confirm that improved renal response comes with better survival outcomes in patients who have baseline renal impairment. Overall, the data suggest that Kd56 (carfilzomib 56 mg/2 plus dexamethasone) has a favourable benefit–risk profile and should be considered standard therapy in patients with RRMM, regardless of baseline renal function [26]. Based on the available pharmacokinetic data, no adjustment of the initial carfilzomib dose is recommended for patients with mild, moderate or severe baseline renal impairment, or for patients receiving chronic dialysis therapy. Pomalidomide/carfilzomib plus dexamethasone appeared to achieve a better response rate than pomalidomide/carfilzomib administered as a single agent. The combination of pomalidomide, carfilzomib and dexamethasone resulted in a much higher response rate than the therapy combining pomalidomide with dexamethasone [27].

#### 3.2.3. Ixazomib

Ixazomib is an oral, highly selective and reversible proteasome inhibitor. Ixazomib preferentially binds and inhibits the chymotrypsin-like activity of the beta-5-subunit of 20S proteasome, which leads to the disruption of cellular regulatory mechanisms, which in turn inhibits cell growth and survival pathways and results in the induction of apoptosis [28]. Based on the PK and safety results, a reduced ixazomib dose of 3 mg (on days 1, 8 and 15 of the 28-day cycles) in MM patients with severe renal insufficiency or ESRD requiring haemodialysis is recommended, compared to the recommended standard 4 mg dose for patients with normal renal function or mild or moderate RI. In patients requiring haemodialysis with ESRD, ixazomib can be administered regardless of the time of dialysis [29].

#### 3.2.4. Marizomib

Marizomib is a β-lactone-γ-lactam proteasome inhibitor derived from the marine actinobacterium Salinispora tropica [30]. In addition to inducing apoptosis, marizomib regulates various signal pathways for cell growth and survival in MM cells. In reality, the initial justification for the therapeutic approach of proteasome inhibitors as anticancer drugs partly relied on their ability to inhibit growth and survival signals via NF-κB [31]. As with bortezomib, marizomib targets NF-κB; what is important is that marizomib is a more potent inhibitor of NF-κB and related cytokine secretion than bortezomib [32]. Phase one studies showed relatively low toxicities and no indication of any neuropathy or thrombocytopenia. In a dose-escalation study comprising 15 patients with relapsed/refractory MM who were being treated with marizomib in monotherapy, three patients resistant to bortezomib responded at least partially [33]. Further studies are investigating the administration of marizomib in patients with RRMM.

#### 3.2.5. Oprozomib

Oprozomib is an oral, irreversible PI derived from carfilzomib that demonstrates an effectiveness in cytotoxicity tests similar to that of carfilzomib. As with bortezomib and carfilzomib, it is highly selective for the CT-L (ß5) subunit of 20S proteasome [24].

The results indicate that therapy with oprozomib and dexamethasone improves gastrointestinal tolerability in patients with relapsed and/or refractory MM relative to single-agent oprozomib [34].

### 3.3. Monoclonal Antibodies Targeting CD38 

#### 3.3.1. Daratumumab

Daratumumab is a human IgG1κ monoclonal antibody that binds to a unique CD38 epitope [35]. Except for a few case reports, there are no studies on daratumumab in patients with severe renal insufficiency (estimated glomerular filtration rate (eGFR) < 15 mL/min/1.73 m^2^). A study by Kuzume A et al. reviewed 13 consecutive patients with MM who had been admitted to the Kameda Medical Center between June 2017 and November 2020 with severe renal failure (≤15 mL/min/1.73 m^2^) and received at least eight daratumumab doses (16 mg/kg). Daratumumab kills myeloma cells shortly after infusion through a variety of mechanisms, including complement-mediated cytotoxicity, antibody-dependent cytotoxicity and antibody-dependent phagocytosis. The adverse effects of daratumumab in patients with severe renal insufficiency are similar to those in patients without renal insufficiency [36,37]. In the DARE study, the administration of daratumumab in combination with dexamethasone to patients with RRMM and severe RI, including dialysis patients, induced a rapid hematological response and, in 17.1% of the patients, a strong renal response. What is important is that no new safety signals were observed, and daratumumab can be safely administered to patients with severe RI or dialysis patients [38].

#### 3.3.2. Isatuximab

Isatuximab (Isa) is a monoclonal IgG1 antibody that targets a specific epitope on CD38 using several different mechanisms of action against multiple myeloma. The prespecified subgroup analysis of ICARIA-MM—the first randomised phase three study to prove a significant survival benefit of an anti-CD38 therapy (isatuximab in combination with pomalidomide) in contrast to pomalidomide in heavily pretreated patients with RRMM—has shown that isatuximab-pomalidomide is also effective with a manageable safety profile in patients with renal insufficiency. Compared to pomalidomide, isatuximab-pomalidomide increased the share of RRMM patients with renal insufficiency who achieved both complete (71.9% with Isa-Pd, 38.1% with Pd) and sustained (≥60 days; 31.3% vs. 19.0%) renal responses, supporting the role of Isa-Pd in achieving durable RI reversal [39].

### 3.4. Monoclonal Antibodies Targeting CS1 (SLAMF7)

#### Elotuzumab

Elotuzumab is a humanised immunostimulatory monoclonal IgG1 antibody that targets the signalling lymphocyte activation molecule F7 (SLAMF7, also referred to as CS1), a glycoprotein that is expressed in myeloma and natural killer cells but not in normal tissue [40]. Elotuzumab in combination with lenalidomide and dexamethasone gave rise to an overall response of 75% in six patients, 67% in six patients and 56% in five patients in the NRF group (normal renal function), the SRI group (severe renal impairment) and the ESRD group (end-stage renal disease), respectively. A VGPR or better was observed in three (38%), five (56%) and one (11%) patients in the NRF, SRI and ESRD groups, respectively. The results of the study support the administration of elotuzumab without dose adjustment for the treatment of MM patients with renal impairment. Elotuzumab combined with lenalidomide and dexamethasone was well-tolerated by MM patients with renal impairment, including patients with terminal renal failure, and effective; this combination could be considered a new therapy option for these patient populations [41].

### 3.5. Monoclonal Antibodies Targeting BCMA

#### 3.5.1. Belantamab Mafoditin

Belantamab mafodotin (GSK2857916) is a first-in-class anti-BCMA immunoconjugate with an afucosylated, humanised IgG1 anti-BCMA monoclonal antibody conjugated by a protease-resistant maleimidocaproyl linker to a microtubule-disrupting agent, monomethyl auristatin F (MMAF) [42]. The DREAMM-2 study showed that, in small, prespecified sub-cohorts of patients with moderate renal impairment or cytogenetic high-risk features, a similar proportion of patients achieved the same overall response as the overall population (although a smaller percentage of patients with extramedullary disease appeared to achieve an overall response than in the overall population), which suggests that these high-risk patients respond equally well to belantamab mafodotin. A longer follow-up is required to determine whether these results lead to an overall survival benefit [43]. In patients with mild or moderate renal impairment (eGRF > 30 mL/min) no dose adjustment is necessary. Insufficient data are available for patients with severe renal impairment to support any dose recommendation.

#### 3.5.2. Anti-BCMA CAR T-Cell Therapy bb2121 (Idecabtagene Vicleucel)

T-cell therapy using chimeric antigen receptors (CARs) has proven to be a new therapy with the potential of ensuring long-term disease control in patients with some types of haematological cancer; anti-CD19 CAR T-cell therapies have been shown to be effective in patients with leukaemia or lymphomas [44,45]. The B-cell maturation antigen (BCMA) is a member of the tumour necrosis factor superfamily of proteins that is expressed primarily by malignant and normal plasma cells as well as some mature B-cells. This provides a potential point of intervention in patients with multiple myeloma [46]. Idecabtagene-vicleucel induces responses in most heavily pretreated patients with refractory and relapsed myeloma; in 26% of the patients undergoing treatment an MRD-negative status was achieved. Nearly all patients exhibited grade three or four toxic effects, most commonly haematologic toxic effects and cytokine release syndrome [47]. So far, no studies have been conducted on hepatic and renal insufficiency with idecabtagene vicleucel.

#### 3.5.3. Monoclonal Antibodies Targeting APRIL

APRIL (a proliferation-inducing ligand) is a member of the tumour necrosis factor family and one of two ligands for BCMA [48]. APRIL is produced by cells in the bone marrow, including myeloid cells, osteoclasts and DCs. APRIL can promote the survival of malignant plasma cells and protect myeloma cell lines from apoptosis after IL-6 deprivation. APRIL also promotes cell cycle progression in myeloma cells [49]. An antagonistic anti-APRIL antibody, hAPRIL01A (01A), has been generated to block APRIL binding to BCMA and TACI [50]. Higher APRIL concentrations can promote resistance to lenalidomide, bortezomib and other standard drugs, encourage the expression of programmed cell death for ligands 1 PD-L1, interleukin (IL)-10 and TGFβ on BCMA+ tumour cells as well as the creation of an immunosuppressive niche that favours tumour cells [49]. Clinical studies evaluating the safety and effectiveness of monoclonal antibodies that target APRIL are continuing to recruit MM patients.

### 3.6. Monoclonal Antibodies Targeting Immune Checkpoints

#### PD-L1

In multiple myeloma, programmed death receptor 1 (PD-1) is strongly expressed, which suggests that a therapy targeting it or its ligands (PD-L1 or PD-L2) would be an effective strategy [51]. Based on promising phase two data on the combination of pembrolizumab with pom/dex and len/dex in RRMM patients, three clinical phase three studies—KEYNOTE-183, KEYNOTE-185 and CHECKMATE-602—examined pomalidomide/dexamethasone with or without pembrolizumab in RRMM patients, lenalidomide/dexamethasone with and without pembrolizumab in newly diagnosed MM patients not eligible for autografts in addition to nivolumab plus pomalidomide-dexamethasone versus pomalidomide-dexamethasone alone or pomalidomide/dexamethasone/elotuzumab/nivolumab in patients with RRMM, respectively. All studies were put on hold early due to increased mortality among the patients who were administered pembrolizumab, with a hazard ratio for mortality of 1.61 in KEYNOTE-183 and 2.06 in KEYNOTE-185, or nivolumab in CHECKMATE-602 (the hazard ratio for mortality was 1.19). Furthermore, the ORRs did not increase when pembrolizumab or nivolumab were added [9,52,53]. These results have raised serious doubts as to the benefit in MM patients, at least in combination with the immunomodulatory agents [54]. A dose adjustment is not required in patients with mild or moderate renal impairment. Pembrolizumab was not studied in patients with severe renal impairment. In a case study, a normal dose of pembrolizumab was administered to a dialysis patient [55]. According to data on population pharmacokinetics (PKs), no dose adjustment is required when administering nivolumab to patients with mild or moderate renal insufficiency. Data on patients with severe renal insufficiency are limited, and no conclusions can be drawn for this population based on the data available. Some case reports indicate that normal nivolumab dosing in dialysis patients is safe [56].

### 3.7. DNA-Damaging Agents

#### Melflufen

Melphalan flufenamide ethyl ester (melflufen) is a peptidase-potentiated alkylating agent which appears to be more efficient than melphalan [57]. Melflufen may have a different mechanism of action than other alkylating agents. Melflufen more effectively induced cell death in TP53-mutated cell lines, for example, and in the cells of patients with TP53-mutated RRMM than melphalan, which indicates that the cytotoxicity mechanisms of melflufen—but not of the other alkylating agents—is independent of the p53 function [57,58]. The results of the HORIZON study suggest that melflufen has the potential of being an important therapeutic option in RRMM patients, as it offers a novel mechanism of action, clinically significant effectiveness and controllable safety in heavily pretreated patients when combined with dexamethasone [59]. The results of the BRIDGE (OP-107) study will be of significance for patients with multiple myeloma and renal insufficiency [60]. As melflufen is quickly and entirely metabolised by aminopeptidases, and because melphalan is mainly eliminated from the plasma by spontaneous hydrolysis—a process that is independent of renal function or liver metabolism—the supposition is that renal impairment has no influence on the pharmacokinetics of melflufen and only little influence on the pharmacokinetics of melphalan [61,62].

### 3.8. Inhibitors of BCL2 Family Proteins

#### Venetoclax

Venetoclax is a highly selective, potent, oral BCL-2 inhibitor that induces apoptosis in multiple myeloma cell lines and primary multiple myeloma cells [63]. Preclinical studies have shown that both glucocorticoid dexamethasone and the proteasome inhibitor bortezomib can increase BCL-2 dependence in multiple myeloma cells by shuttling proapoptotic proteins from MCL-1 to BCL-2, simultaneously reducing BCL-XL expression and MCL-1 activity through the up-regulation of noxa (PMAIP1) [64,65]. The results of the BELLINI study show that, in contrast to the placebo combined with bortezomib and dexamethasone, venetoclax combined with bortezomib and dexamethasone improves progression-free survival, the overall response rate and the rate of very good partial response or better, although the increased risk of mortality indicates an unfavourable risk–benefit profile for biologically unselected patients with relapsed or refractory multiple myeloma. The fact that patients with t(11;14) and patients with higher BCL2 expression appear to have a more favourable benefit–risk profile than patients without t(11;14) and with lower BCL2 expression suggests that a biomarker-driven approach may be appropriate for the administration of venetoclax in multiple myeloma patients [66]. In patients with renal impairment (CrCl < 80 mL/min), more intensive prophylaxis and monitoring may be necessary in order to reduce the TLS risk at initiation and during the dose titration phase. In patients with mild, moderate or severe renal impairment (CrCl ≥ 15 mL/min and <90 mL/min), no dose adjustment is necessary. Venetoclax should only be administered to patients with severe renal impairment (CrCl ≥ 15 mL/min und < 30 mL/min) if the benefits outweigh the risks, and the patients must be closely monitored for signs of toxicity because of an elevated tumour lysis syndrome risk.

### 3.9. Epigenetic Inhibitors

#### Histone Deacetylase (HDAC, Panobinostat)

Panobinostat is a histone deacetylase (HDAC) inhibitor that inhibits all HDAC proteins of the HDAC families I, II and IV in the nanomolar range in vitro [67]. Panobinostat is administered clinically in combination with bortezomib or lenalidomide and dexamethasone in patients with relapsed and refractory MM. However, single-agent panobinostat lacks any therapeutic activity. A challenge resulting from pan-HDAC inhibition is the lack of specificity and the observed adverse effects that frequently lead to suspension of the treatment [68]. Beider et al. found that the sensitivity of MM cells and primary MM cells to panobinostat comes with reduced CXCR4 expression, whereas CXCR4 overexpression increased their resistance to Panobinostat [69]. In patients with mild to severe renal impairment, the plasma exposure of panobinostat is unchanged. As a result, an adjustment of the initial dose is unnecessary. The administration of panobinostat to patients with end-stage renal failure (ESRD) or dialysis patients has not been studied. Therefore, the use of panobinostat in patients with GFR < 15 mL/min and in dialysis patients is not recommended.

### 3.10. Inhibitors of Nuclear Cytoplasmic Transport Receptor (XPO1 Inhibitor)

#### Selinexor

Selinexor is an oral, slowly reversible, covalent inhibitor of XPO1-mediated nuclear export. The treatment of cancer cells with selinexor encourages the nuclear retention of TSPs (p53, Rb, FOXO1, survivin and IkB) and blocks the export of eIF4E-bound oncoprotein mRNAs (c-Myc, cyclin D1, Bcl-6, Mdm2 and Pim), resulting in growth inhibition and apoptosis [70]. Selinexor showed promising effectiveness in combination with other antimyeloma therapies, including corticosteroids, IMiDs and anti-CD38 antibodies. In a first phase one study on humans, selinexor plus low-dose dexamethasone showed an overall response rate (ORR) of up to 50% at the recommended phase two dose in patients with heavily pretreated multiple myeloma [71]. Selinexor plus low-dose bortezomib and dexamethasone demonstrate an even better ORR of 63%. The median progression-free survival of all patients was 9.0 months; 17.8 months for non-refractory PI and 6.1 months for refractory PI. The SVd treatment of patients with relapsed or refractory MM, including bortezomib-refractory MM, leads to high response rates without unexpected adverse effects [72]. No adjustment of the selinexor dose is necessary in patients with mild, moderate or severe renal impairment. No data are available for patients with terminal renal insufficiency or haemodialysis. Therefore, no dose recommendation can be made.

## 4. Treatment of Relapsed and Refractory Myeloma in Patients with Renal Insufficiency after First- and Second-Line Therapy

Advances in treatment, including the introduction of immunomodulatory drugs, proteasome inhibitors and monoclonal antibodies, have prolonged survival among multiple myeloma patients [73]. In RRMM disease, many studies comparing the triplet regimen with the doublet regimen have shown a benefit in progression-free survival (PFS), and the ASPIRE12 and ELOQUENT-213 studies have demonstrated overall survival with a toxicity profile that is, by and large, manageable. Therefore, at the time of relapse, a triplet therapy is recommended for fit patients. In frail patients or patients whose treatment objectives coincide with those of low-toxicity regimens, doublet or triplet regimens with a reduced dose can be considered [74,75].

The choice of therapy during the first relapse following first-line therapy depends on whether the disease is refractory to lenalidomide or not. What is likewise important is whether the relapse is a multiple myeloma relapse with daratumumab therapy [76]. The choice of therapy also depends on the comorbidities, including renal insufficiency. When choosing the combined therapy, the effectiveness of the selected therapy in the event of renal insufficiency must be taken into account. In patients with their first RRMM relapse and renal insufficiency, triplet therapy with a CD38 antibody should be initiated without undue delay due to the aggressive nature of the disease (only if the patient has not previously received CD38 antibodies). Daratumumab plus lenalidomide plus dexamethasone is the most effective combination available for a first-time myeloma relapse that is not refractory to lenalidomide [77]. In the POLLUX study, daratumumab plus lenalidomide plus dexamethasone significantly prolonged progression-free survival in the intention-to-treat population versus lenalidomide plus dexamethasone (median 45.8 months vs. 17.5 months; HR 0.43 (95% CI 0.35–0.54); and *p* < 0.0001) after a median follow-up of 51.3 months [78]. In lenalidomide-refractory RRMM, daratumumab with bortezomib and dexamethasone can be used. In the CASTOR study, bortezomib plus dexamethasone was compared to daratumumab plus bortezomib plus dexamethasone in patients with relapsed multiple myeloma who had received at least one previous therapy line. In all patients, the triplet combination was associated with significantly longer progression-free survival (median not reached vs. 7.2 months; HR 0.39 (95% CI 0.28–0.53); and *p* < 0.001) [79]. For this reason, it makes sense to switch to daratumumab plus carfilzomib plus dexamethasone (KdD) for RRMM patients who are refractory or not refractory to lenalidomide. As opposed to carfilzomib plus dexamethasone (Kd), daratumumab plus carfilzomib plus dexamethasone significantly prolonged progression-free survival in patients with relapsed or refractory multiple myeloma and was associated with a favourable benefit–risk profile. After a median follow-up of approximately 17 months, median progression-free survival in the KdD group was not achieved versus 15.8 months in the Kd group (hazard ratio 0.63; 95% CI 0.46–0.85; and *p* = 0.0027). The median treatment period was longer in the KdD group than in the Kd group (70.1 vs. 40.3 weeks). KdD showed a clinical advantage over Kd in that the risk of progression or death was reduced by 36% in patients with prior proteasome inhibitor exposure, including bortezomib [80]. Aside from daratumumab, isatuximab is the next anti-CD38 antibody for the treatment of patients with RRMM after one to three therapy lines. Isatuximab plus carfilzomib plus dexamethasone was superior to carfilzomib plus dexamethasone in terms of progression-free survival, both in patients with prior lenalidomide exposure (HR 0.50 (95% CI 0.29–0.87); *p* value not available) and in lenalidomide-refractory patients (HR 0.60 (95% CI 0.34–1.06); *p* value not available) [81].

A number of protocols with daratumumab in first-line therapy have now been approved, including D-VMP and D-Rd, greatly enriching the first-line therapy options available for MM patients. So far, there is no data supporting a renewed treatment with daratumumab in second-line therapy, and a salvage therapy with isatuximab in patients who progress under daratumumab is presumably not a suitable option since both antibodies target the same antigen (CF38). A suitable option would be carfilzomib plus lenalidomide plus dexamethasone for fit patients over 65 years of age in this setting, but for frail patients or patients over 75 years of age, dexamethasone in combination with ixazomib or elotuzumab could be the best option after progression under daratumumab plus bortezomib plus melphalan plus prednisone [76].

In patients suffering an MM relapse after treatment with bortezomib and lenalidomide, pomalidomide plus dexamethasone was considered standard therapy on the basis of the results of the randomised study MM-003 [82].

Another therapy option in RRMM patients with renal insufficiency who are refractory to both bortezomib and lenalidomide is the administration of pomalidomide plus carfilzomib plus dexamethasone (KPd). The EMN011/HO114 study shows that KPd is a practicable, effective and safe triple-drug regimen for RRMM patients who have previously undergone treatment and/or are refractory to bortezomib and lenalidomide. An overall response rate of 87% including 31% CR/sCR is clinically relevant in this population [83]. In patients with severe renal insufficiency, including dialysis, pomalidomide and carfilzomib doses do not need to be reduced.

## 5. Treatment of Relapsed and Refractory Myeloma with Renal Insufficiency after Three or More Therapy Lines

When the third or further relapse occurs, all the drugs previously considered but not used for the first or second relapse can again be taken into consideration. In the ELOQUENT-3 study, 50 patients who had received at least two previous therapy lines were randomly assigned either elotuzumab plus pomalidomide plus dexamethasone (*n* = 60) or pomalidomide plus dexamethasone (*n* = 57). Median progression-free survival with elotuzumab plus pomalidomide and dexamethasone was 10.3 months and the overall response rate amounted to 53%, which alone demonstrates the superiority of this treatment over pomalidomide and dexamethasone (4.7 months in the group with pomalidomide plus dexamethasone, HR 0.54 (95% CI 0.34–0.86); *p* = 0.0080) [84]. Another study proved a median PFS of 6.4 months, with PRS rates after 12 and 18 months being similar to those reported in a current update of the ELOQUENT-3 study [85]. An adjustment of the elotuzumab and pomalidomide doses is not required in patients with severe renal insufficiency, including kidney disease in the end stage. The results showed a median progression-free survival benefit with daratumumab plus pomalidomide plus dexamethasone over pomalidomide plus dexamethasone. With a median follow-up of 16.9 months (IQR 14.4–20.6), progression-free survival in the group receiving daratumumab plus pomalidomide and dexamethasone improved more than in the group receiving pomalidomide and dexamethasone (median 12.4 months (95% CI 8.3–19.3) vs. 6.9 months (5.5–9.3), hazard ratio 0.63 (95% CI 0.47–0.85) and two-sided *p* = 0.0018) [86]. There is a simple and affordable option to improve the results of pomalidomide plus dexamethasone when anti-CD-38 antibodies or elotuzumab is not available, and that is the addition of cyclophosphamide to pomalidomide and dexamethasone. The study has shown that pomalidomide in combination with dexamethasone and oral weekly cyclophosphamide in patients with lenalidomide-refractory multiple myeloma leads to a better response rate than pomalidomide and dexamethasone. In addition, the combination of orally administered cyclophosphamide once weekly with pomalidomide and dexamethasone was well tolerated, with only a moderate increase in hematological toxicity that failed to reach any statistically relevant level. Patient without high-risk cytogenetics appear to benefit more from a combination of pomalidomide, cyclophosphamide and dexamethasone, whereas patients with high-risk cytogenetics tend to have worse OS with this combination than with pomalidomide and dexamethasone [87]. This combination is particularly appropriate when daratumumab, isatuximab or elotuzumab are not available and the patient does not have any high-risk cytogenetics. Cyclophosphamide can be used in patients with severe renal insufficiency, whereas a 50% dose reduction is recommended when the GFR is below 10 mL/min. In patients requiring dialysis, the period between the administration of cyclophosphamide and dialysis should always be the same if possible. 

## 6. Further Therapy Options for Severe Pretreated Patients with RRMM and Renal Insufficiency

Patients with multiple myeloma that is refractory to proteasome inhibitors, immunomodulators and anti-CD38 antibodies have a very poor prognosis. One study showed that these patients have a median overall survival of only 5.6 months [88]. The prognosis for patients also suffering from renal insufficiency was particularly poor. However, new drugs have been developed in recent years that have already undergone phase one and two clinical trials. These drugs have different mechanisms of action and pharmacokinetics, therefore their use in RRMM patients with renal insufficiency must be interpreted differently.

Belantamab mafodotin is an anti-BCMA antibody–drug conjugate that contains monomethyl auristatin F. In the phase two study DREAMM-260, 196 patients with triple-class refractory multiple myeloma were administered two different doses of belantamab mafodotin (2.5 mg/kg (*n* = 97) or 3.4 mg/kg (*n* = 99)). The overall response rate was 31% in the group with the 2.5 mg/kg dose and 34% in the group that received the 3.4 mg/kg dose. Median progression-free survival was 2.9 months in the 2.5 mg/kg group and 4.9 months in the 3.4 mg/kg group, but no overall survival data were available at the time of publication in December 2019. The DREAMM-2 study showed that in small, pre-defined sub-cohorts of patients with moderate renal impairment or high-risk cytogenetic factors, the proportion of patients who achieved an overall response was similar to that in the overall population [43]. The phase two HORIZON study investigated the effectiveness of melflufen plus dexamethasone in patients with relapsed and refractory multiple myeloma (RRMM). Out of 157 patients (median age 65 years; median five previous therapy lines) that accepted and were treated in the study, 119 patients (76%) had triple-class refractory disease, 55 (35%) had extramedullary disease and 92 (59%) were refractory to previous alkylator therapy. The overall response rate was 29% in the overall population with 26% in the triple-class refractory population. The median response period in the overall population was 5.5 months, whereas median progression-free survival was 4.2 months and median overall survival was 11.6 months in a median follow-up period of 14 months [89]. As melflufen is quickly and entirely metabolised by aminopeptidases and because melphalan is mainly eliminated from the plasma by spontaneous hydrolysis—a process that is independent of renal function or liver metabolism—the supposition is that renal impairment has no influence on the pharmacokinetics of melflufen and only little influence on the pharmacokinetics of melphalan [61]. The data from other studies is missing and will be assessed in future studies.

Furthermore, there are no tolerability studies for these new drugs (selinexor, panobinostat, venetoclax, monoclonal antibodies targeting APRIL or anti-BCMA CAR T-cell therapy bb2121) in patients with mild renal insufficiency (GFR 60–30 mL/min.), severe renal insufficiency (GFR 30–15 mL/min.) and end-stage renal disease (GFR < 15 mL/min. up to dialysis). The use of new drugs in patients with end-stage renal disease (ESRD) or dialysis patients has not yet been studied. Therefore, the administration of new drugs in patients is currently not recommended.

## 7. Conclusions

New drugs have helped to widen the treatment options and prognosis for patients with renal impairment and refractory/relapsed multiple myeloma, since dose adjustments are unnecessary with carfilzomib as well as with panobinostat, elotuzumab, pomalidomide or daratumumab in patients with renal impairment. In the meantime, several new substances have been approved for the treatment of refractory/relapsed multiple myeloma. The most important result of several studies is that the effectiveness and safety of novel agents in patients with RI is similar to their effectiveness and safety in patients with normal renal function. However, the approval studies did not include patients with severe RI or dialysis patients. As a result, further studies that include patients with severe or terminal renal insufficiency are needed in order to verify the effectiveness of these novel agents in patients and to allow conclusions to be drawn as to the optimal dosing of these drugs in patients with severe and terminal renal insufficiency.

## Figures and Tables

**Table 1:** New drugs in the treatment of patients with RRMM and renal and hepatic insufficiency.

Drugs	Dose Adjustment Renal Insufficiency (RI)	Dose Adjustment Hepatic Insufficiency
Lenalidomide	GFR 30–50 mL/min.: 10 mg/day GFR < 30 mL/min.: 7.5 mg/day Terminal RI: 5 mg/day On dialysis days, the dose should be administered post-dialysis	N.A.
Pomalidomide	No dose adjustment On dialysis days, the dose should be administered post-dialysis	No dose adjustment
Iberdomide	N.A.	N.A.
Carfilzomib	No dose adjustment On dialysis days, the dose should be administered post-dialysis	Mild/moderate hepatic insufficiency: no dose reduction Dose reduction recommended if liver transaminase levels are elevated
Ixazomib	GFR 60–30 mL/min.: no dose adjustment GFR < 30 mL/min. until dialysis: dose reduction to 3 mg Time of administration does not depend on time of dialysis	Mild hepatic insufficiency: no dose adjustment Moderate/severe hepatic insufficiency: dose reduction to 3 mg
Marizomib and oprozomib	N.A.	N.A.
Daratumumab	No dose adjustment	No dose adjustment
Isatuximab	Mild to severe RI: no dose adjustment	No dose adjustment
Elotuzumab	No dose adjustment	Mild hepatic insufficiency: no dose adjustment Moderate/severe hepatic insufficiency: N.A.
Blentamab mafoditin		Bilirubin > ULN to < 1.5 x ULN or AST > ULN: no dose adjustment
	EGFR 60–30 mL/min.: no dose adjustment	Moderate and severe hepatic insufficiency: N.A.
	EGFR < 30 mL/min.: N.A.	
Idecabtagene vicleucel	N.A.	N.A.
Melflufen and selinexor	N.A.	N.A.
Venetoclax	GFR 90–30 mL/min.: no dose adjustment	Moderate hepatic insufficiency: caution in the dose titration phase due to TLS (tumour lysis syndrome)
	GFR < 30 mL/min. and dialysis: no therapy recommended	
